# Factor structure and psychometric properties of the Chinese version of the Odor Awareness Scale

**DOI:** 10.3389/fpsyt.2023.1228179

**Published:** 2023-07-27

**Authors:** Binfeng Zhang, Xiuxia Li, Peixuan Tan, Yuxing Liu, Wanyong He, Lu Wang, Shuling Huang, Guanghui Nie

**Affiliations:** Department of Psychology, Guangxi Medical University, Nanning, China

**Keywords:** odor awareness, factor analysis, Chinese, olfactory, affective symptoms

## Abstract

**Background:**

The Odor Awareness Scale (OAS) is a questionnaire that assesses individual differences in awareness of odors in the surrounding environment, which has been shown to be associated with affective symptoms in recent researches. To further research, A Chinese version of the OAS needs to be introduced.

**Objective:**

To investigate the factor structure and validate the psychometric properties of the OAS.

**Methods:**

A total of 978 participants from college were randomly allocated into two groups for exploratory factor analysis (EFA) and confirmatory factor analysis (CFA), respectively. Additionally, the study entailed item analysis and scrutinized internal consistency reliability, test-retest reliability, and concurrent validity. Test-retest reliability was assessed by having 214 participants complete the OAS twice at a one-week interval. Concurrent validity was measured using the Body Odor Sniffing Questionnaire (BOSQ), the Generalized Anxiety Disorder Scale (GAD-7), and the Toronto Alexithymia Scale (TAS-20).

**Results:**

EFA identified three factors that best fit the data: odor sensitivity, odor impact, and odor attention. CFA validated a second-order factor model, yielding good fit indices: $\chi^{2}$ / Df = 2.326, RMSEA = 0.052, CFI = 0.911, TLI = 0.900, SRMR = 0.053. The final version of the OAS comprised 27 items and exhibited a commendable internal consistency reliability (Cronbach’s α = 0.913), and a good test-retest reliability, as evidenced by the high Pearson correlation coefficient (*r* = 0.940) and intraclass correlation coefficient (ICC = 0.940). The OAS was significantly correlated with BOSQ (*r* = 0.416), GAD-7 (*r* = 0.155), and TAS-20 (*r* = −0.081).

**Conclusion:**

The Chinese version of the OAS demonstrated robust reliability and validity, rendering it a valuable instrument for evaluating odor awareness in the Chinese population.

## 1. Introduction

Olfaction plays an important role in human activity. Our environment is replete with various stimuli that engage various sensory modalities, and among them, olfactory stimuli wield significant influence over behavior, cognition, and emotion (1–5). For instance, astute retailers arrange pleasant fragrances to promote sales (6, 7), while the perception of body odor in romantic encounters influences partner selection (8). However, considerable individual differences can be found in the degree of impact of odors on people and their awareness of them (3, 9). Some people process olfactory information on a subconscious level. Conversely, for others, odors stand out in the olfactory environment. These individuals swiftly detect the presence of odors in their surroundings and spontaneously offer remarks, relying on past experiences to actively seek pleasant olfactory stimuli and avoid unnecessary and potentially unpleasant ones (10). Such variations might stem from genetically determined psychological and biological propensities, as well as the impacts of individual experiences, gender, health status, developmental factors, or other broader influences on capabilities and cultural predispositions (11).

Tools for measuring odors are necessary because of the effects of odors on our daily lives and the existence of individual differences. Such tools can help better understand the relationship between olfactory function and affect, cognition, and behaviors. Many questionnaires are available to measure an individual’s olfactory information and provide cognitive content, such as attitude, ability, and awareness of odor (3, 9, 12–16). For example, the Affective Impact of Odors Questionnaire measures how odors affect an individual’s liking and memory of places, things, and people (3). The Odors in Everyday Life Questionnaire investigates the perceptual role of odors in environmental assessment, everyday life practices, sex, social relationships, and memory (12). The Children’s Olfactory Behavior in Everyday Life questionnaire measures children’s active olfactory search, perception, and affective responses to food, people, and environmental odors (13).

One widely used olfactory measurement scale for adults is the Odor Awareness Scale (OAS) (9). Odor awareness, in this sense, refers to the extent to which an individual is inclined to receive olfactory stimuli and rely on them to guide attitudes and actions (9, 10, 17). The OAS measures an individual’s tendency to perceive, attend to, or value environmental odors, encompassing issues related to food, beverages, environments and man-made sources (9). The original scale was developed based on a sample of college students (23.1 ± 5.8 years) and has two factors (i.e., positive factor and negative factor). And then, a Spanish version of the OAS, based on a sample of adults (30.81 ± 7.27 years), has been revised (18). And exploratory factor analysis (EFA) was conducted using the principal axis factoring method, which extracted one dimension with a Cronbach’s α of 0.906, different from the two factors hypothesized by the original scale. Because there is currently no Chinese version of the OAS, this study aims to translate, revise and validate the OAS for use in the Chinese population.

Odor awareness is closely related to olfactory performance in individuals with varying levels of odor awareness. Individuals with low odor awareness have been associated with lower olfactory performance, such as olfactory thresholds, recognition, and discrimination, than those with high odor awareness (9, 10). Additionally, individuals with high odor awareness report better olfactory recognition memory and spontaneously identify more odors than those with low odor awareness (19). Odor awareness is crucial to personal olfactory experience and can indirectly represent actual olfactory performance (9, 10). The limbic system is closely linked to olfaction and emotion, with anatomical overlap between the olfactory system and emotion-related regions in the limbic system (20–22). This link allows the limbic system to process emotional signals conveyed by other sensory modalities while also processing olfactory signals, providing a basis for the interaction between olfaction and emotion (23), which might be responsible for the close correlation between olfactory dysfunction and affective disorders (24–27). And that may be the reason why odor awareness is associated with affective disorder or symptoms.

Studies have shown that individuals with panic disorder have higher odor awareness than healthy controls (28, 29). Individuals with anxiety symptoms and neurotic personality traits have higher odor sensitivity and reactivity than healthy individuals (30–35). In a study of a non-clinical population, anxiety symptoms positively predicted odor awareness. In contrast, alexithymia negatively predicted odor awareness, and the interaction between alexithymia and social anxiety symptoms also significantly influenced odor awareness (17). Therefore, odor awareness may serve as a tool for screening affective symptoms and promoting the prevention and treatment of affective disorders. Based on the relationship between odor awareness and anxiety symptoms and alexithymia, this study utilized the Generalized Anxiety Disorder Scale (GAD-7) and the Toronto Alexithymia Scale (TAS-20) as validity measures for the Chinese version of the OAS among Chinese college students.

This study aims to explore the factor structure of the Chinese version of the OAS and its psychometric characteristics, including construct validity, internal consistency reliability, test-retest reliability, and concurrent validity.

## 2. Materials and methods

### 2.1. Participants

We recruited participants from Guangxi Medical University in China by distributing paper questionnaires in classrooms anonymously. Students voluntarily participated in the study through inquiry, and those who completed the questionnaire would receive five points for their regular grade. The eligibility criteria for participants were as follows: (1) native citizens and (2) aged between 18 and 45 years old to reduce olfactory variability associated with aging. Exclusion criteria were as follows: (1) acute respiratory infections; (2) chronic obstructive pulmonary disease or nasal surgery; (3) other neurological diseases that affect olfactory function, such as Parkinson’s disease and epilepsy; and (4) unstable mental illnesses such as schizophrenia. A total of 1,000 questionnaires were distributed, with 990 collected. After excluding 7 with missing data and 5 questionnaires that did not pass the lie detection questions, the sample included 978 participants consisting of 270 males and 708 females, whose ages ranged from 18 to 35 (*M* = 21.90, SD = 2.06). The sample was randomly divided into two equal subsamples: 489 data were used for EFA to explore the factor structure of OAS (Sample 1: 131 males and 358 females); the other 489 questionnaires were used for confirmatory factor analysis (CFA) to validate the obtained factor structure from EFA (Sample 2: 139 males and 350 females). Among this sample, 214 participants completed the OAS twice at a one-week interval to measure the test-retest reliability. The implementation of this study followed the principles of the Helsinki Declaration and was approved by the Ethics Committee of Guangxi Medical University.

### 2.2. Measures

#### 2.2.1. The Odor Awareness Scale

The OAS consists of 32 items, with different options for each item (9). Most items use a five-point scale. Items 1–20 are rated from “1-never” to “5-always”; items 21–23 are rated from “1-(almost) no influence” to “5-very much influence”; item 24 is rated from “1-much less sensitive than others” to “5-much more sensitive than others”; item 25 is rated from “1-not annoyed at all” to “5-very annoyed”; items 26–29 are rated from “1-not at all important” to “5-very important”; item 30 is rated from “1-no” to “5-yes”; item 31 is a four-point scale, “1-I will not let the odor of the supermarket affect my shopping, 2-I avoid going to supermarkets with less pleasant odors, 3-I only go there when there is no other choice, 4-I will never go there again”; item 32 is a four-point reverse rating, evaluating the importance of an option (i.e., all-purpose cleaner). The scale has two factors: (1) a positive factor representing pleasant or attractive sources of odors that can be safely approached, and (2) a negative factor representing dangerous or uncomfortable odors that are best avoided. The positive factor includes 11 items with a Cronbach’s α of 0.77, and the negative factor includes 21 items with a Cronbach’s α of 0.80. And CFA has been conducted to test the factor validity of the positive and negative factors separately, which yielded good fit indices. The OAS score is calculated by summing up all items, with a higher score indicating a stronger odor awareness.

We have obtained authorization for translation and revision from the original author of the scale. The original scale was translated into Chinese by a bilingual psychology professor and a graduate student and then distributed to 20 graduate students for preliminary testing. After modifications based on their feedback, the scale was back-translated into English by an English teacher familiar with psychology to ensure fidelity to the original text. For items where ambiguities between the back-translated version and the original arose, a group of two bilingual psychology doctors and seven graduate students discussed and revised them repeatedly, taking into account the cultural background of China, resulting in the final Chinese version of the Odor Awareness Scale.

#### 2.2.2. The Body Odor Sniffing Questionnaire

The Body Odor Sniffing Questionnaire (BOSQ), developed by Li et al. (16), evaluates individuals’ body odor-sniffing behaviors. The BOSQ consists of 19 items divided into three dimensions: other’s body odor, self-common body odor, and self-private body odor. Each item is scored on a Likert scale ranging from 0 (never) to 3 (often), with a higher total score indicating a higher frequency of sniffing one’s own or others’ body odor. In this study, Cronbach’s α coefficient for the BOSQ was 0.892.

#### 2.2.3. The Toronto Alexithymia Scale

The Toronto Alexithymia Scale (TAS-20) was developed by Bagby et al. (36) to measure the severity of alexithymia. The scale consists of 20 items divided into three dimensions: difficulty identifying feelings and distinguishing them from the bodily sensations of emotions, difficulty describing feelings to others, and an externally oriented cognitive thinking style. The scale is scored using a 5-point Likert scale (1 = strongly disagree to 5 = strongly agree), with five items being reverse scored (i.e., items 4, 5, 10, 18, and 19). A higher total score indicates a higher level of alexithymia. This study used the Chinese version of TAS-20 translated and revised by Jinyao et al. (37). The Cronbach’s α of TAS-20 was 0.824.

#### 2.2.4. The Generalized Anxiety Disorder Scale

The Generalized Anxiety Disorder Scale (GAD-7), developed by Spitzer et al. (38), is a screening tool used to assess the severity of generalized anxiety symptoms, which is a component of the Patient Health Questionnaire (PHQ). GAD-7 consists of seven items designed to assess how often patients have been bothered by seven problems, including feeling nervous and worried in the past 2 weeks. Response options range from “not at all” to “nearly every day” and are scored as 0, 1, 2, or 3. In this study, we used the Chinese version of GAD-7 translated and revised by Xiaoyan et al. (39). The Cronbach’s α coefficient for GAD-7 was 0.900.

### 2.3. Statistical analysis

Before conducting factor analysis, we first performed item analysis on the entire sample using the item-total correlation and critical ratio method. After the preliminary screening of items, EFA was conducted on Sample 1 using principal component analysis and maximum variance method in SPSS 26.0 to explore the scale’s factor structure. Sample 2 was used for CFA using Mplus 8.3, and maximum likelihood estimation was used to confirm the factor structure obtained from EFA.

In addition, we calculated the internal consistency reliability of the entire sample using Cronbach’s α coefficient. A total of 214 participants were randomly selected from the sample and were asked to complete the questionnaire twice at a one-week interval to assess the test-retest reliability. Test-retest reliability was calculated using the Pearson correlation coefficient and intraclass correlation coefficient (ICC).

## 3. Results

### 3.1. Item analysis

Item-total correlation analysis showed all items were significantly correlated with the scale’s total score, with most items having a Pearson correlation coefficient greater than 0.3, ranging from 0.351 to 0.635. However, the item-total correlation coefficients of item 31 and item 32 were below 0.3, with coefficients of 0.260 and 0.255 respectively, indicating low homogeneity with the scale and thus were removed from the analysis (40, 41). The discriminant validity of each item was examined using the critical ratio method. Two sub-samples were created by dividing the total score into top and bottom 27%, and a *T*-test was conducted for each item between these two groups. The results indicated that the discriminant validity of each item was good, with all *T*-values reaching a significant level (*p* < 0.05).

### 3.2. Exploratory factor analysis

In the exploratory factor analysis with Sample 1, item intercorrelations showed that most items correlated greater than 0.3 with at least one other item, except for item 25, which had correlations of less than 0.3 with all other items and was deemed unsuitable for factor analysis and removed (42). The Kaiser-Meyer-Olkin (KMO) and Bartlett’s sphericity tests were conducted to test if the sample was suitable for the EFA. The KMO value was 0.922, achieving the acceptable range of 0.8–0.9 (43). Bartlett’s test was also significant ( $\chi^{2}$ = 5337.201, *p* < 0.001). These results indicated that the sample was eligible for EFA.

Based on the scree plot, eigenvalues, and cumulative variance percentage, four models were considered: a two-, three-, four-, and five-factor model. To ensure the quality of the scale, the following criteria were used for item selection: (1) items with factor loadings of less than 0.4 were removed gradually, and (2) items with cross-loadings greater than 0.4 were removed progressively (42, 44–46). Because the original scale was designed with a two-factor structure, the two-factor model was analyzed first. However, the original two-factor items were distributed across our two-factor structure. Two items needed to be removed to meet our item selection criteria and achieve a cumulative variance percentage of less than 40%. We also explored three-, four-, and five-factor structures. After orthogonal rotation, the three-factor structure fits the data best. Only item 14 needed to be removed to meet the item selection criteria, and the cumulative variance percentage was 43.774% (see Table 1). Ultimately, 28 items were retained for the confirmatory factor analysis.

**Table 1:** Rotated component matrix of the Chinese version of OAS.

Items		Factor 1	Factor 2	Factor 3
		Odor sensitivity	Odor impact	Odor attention
11.	Are you the first one to smell when the milk is sour?	0.749		
10.	Are you the first one to smell gas?	0.690		
13.	Are you the first one to smell spoilt food in the fridge?	0.661		
12.	Are you the first one to smell a fire, even when the smell only comes from a barbecue or fireplace?	0.656		
8.	Do you notice the smell of people’s breath or sweat?	0.608		
9.	Do you pay attention to the perfume, the aftershave or deodorant other people use?	0.569		
15.	Do you get angry or annoyed by an indistinct or unfamiliar smell in the environment?	0.563		
7.	When an acquaintance smells differently from normal, for example, because of a new perfume, do you immediately notice?	0.524		
17.	Do odors revive strong or vivid memories in you?	0.460		
22.	When someone has an unpleasant body odor, does that make you find him or her unattractive? The body odor		0.661	
27.	How important is it to you that your (future/potential) partner has a pleasant smell?		0.659	
21.	When a room has an unpleasant smell, does it influence your mood?		0.632	
23.	When someone has a pleasant body odor, do you find him or her attractive?		0.586	
30.	You are in a public space sitting close to someone who has an unpleasant smell. Do you look for another seat if possible?		0.582	
26.	How important is it to you that your sheets smell fresh?		0.563	
29.	How important are odors to you in your everyday life?		0.551	
19.	The smell of smoke or food is still lingering in your clothes from the night before. Do you put on new clothes because of the smell?		0.500	
24.	People differ in their sensitivity for odors. An unpleasant smell can leave one person unaffected yet be unbearable to another. How sensitive to odors do you think you are?		0.451	
20.	Does the smell of food sometimes put you off it?		0.437	
16.	Does an unpleasant smell in the environment that will not go away make you anxious?		0.428	
18.	Do you sniff at clothes before you put them on?		0.414	
28.	Nowadays many cultivated flowers no longer have a fragrance. Do you find it important that flowers are fragrant?		0.404	
2.	When someone is busy in the kitchen, do you notice the odor of the food being prepared?			0.800
1.	When you walk through the woods, do you pay attention to the odors surrounding you?			0.765
3.	Do you notice food odors emanating from houses when you are outdoors?			0.742
5.	When you visit someone else’s house, do you notice how it smells?			0.650
6.	Do you sniff at a new book?			0.415
4.	When you are studying, or concentrated in general, do you get distracted by odors in the environment?			0.409
	Eigenvalue	8.705	2.034	1.518
	Variance explained (%)	31.088	7.264	5.421

All items except 14, 25, 31, and 32 were included in final EFA. Factor loadings with absolute value greater than 0.40 are shown. Extraction Method: Principal Component Analysis. Rotation Method: Varimax with Kaiser Normalization.

### 3.3. Confirmatory factor analysis

To further validate the three-factor structure obtained from EFA, we applied CFA using sample 2. We evaluated the goodness of fit of multiple models using several fit indices: (1) $\chi^{2}$ / Df ( $\chi^{2}$ / Df <3); (2) root mean square error of approximation (RMSEA <0.08) and standardized root mean squared residual (SRMR <0.08); and (3) comparison fit index (CFI ≥ 0.90) and Tucker-Lewisfit index (TLI ≥ 0.90). A model with indices meeting these criteria indicates a good fit (47–49). Although the three-factor structure was obtained using orthogonal rotation, which assumes the factors are uncorrelated, we found moderate to high correlations among the three factors in the CFA, indicating that the three factors are not independent and may reflect a higher-order factor. Therefore, we conducted a second-order CFA to validate the three-factor structure and investigate whether the three factors fit the general concept of “odor awareness.”

**Figure 1:**
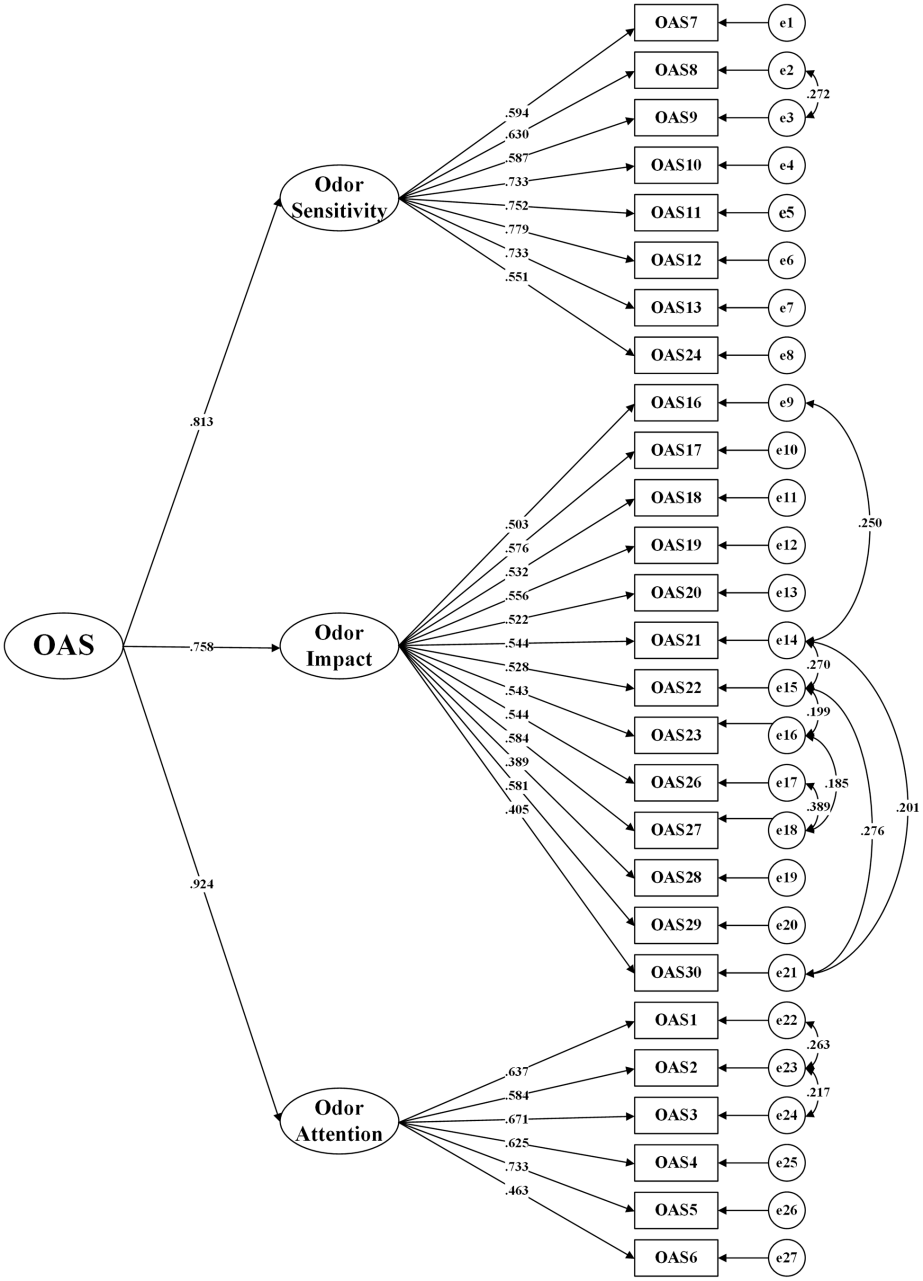
The second-odor model of the OAS with standardized factor loadings except for item 15.

The results of the second-order CFA showed most standard factor loadings were above 0.32, ranging from 0.381 to 0.773, except for item 15, which had a factor loading of 0.284 and an $R^{2}$ of 0.081 less than 10% and was therefore removed (50). Modification indices showed that item 24 would reduce the chi-square value by 51.275 if loaded onto Factor 1, and item 17 would reduce the chi-square value by 34.452 if loaded onto Factor 2, indicating that item 24 may reflect the content of Factor 1 and item 17 may reflect the content of Factor 2. Therefore, we made position adjustments for item 24 and item 17. Finally, we improved the model by adding 10 error covariances and obtained a second-order three-factor model with 27 items that showed satisfactory fit indices: $\chi^{2}$ / Df =2.326, RMSEA = 0.052, CFI = 0.911, TLI = 0.900, SRMR = 0.053 (see Table 2). The standardized factor loadings for all items were greater than or equal to 0.389 (ranging from 0.389 to 0.779) and statistically significant. The standardized factor loadings for the three first-order factors loaded onto the higher-order factor were 0.813 for Factor 1, 0.758 for Factor 2, and 0.924 for Factor 3, all significant (see Figure 1).

**Table 2:** Results of the confirmatory factor analysis.

Fit index	Criteria	Results	Judgment
$\chi^{2}/\mathrm{Df}$	<3	2.326	Yes
CFI	≥0.9	0.911	Yes
TLI	≥0.9	0.900	Yes
SRMR	<0.08	0.053	Yes
RMSEA	<0.08	0.052	Yes
90% CI of RMSEA	(0.047, 0.057)		

CFI, comparative fit index; TLI, Tucker-Lewis index; RMSEA, root mean square error of approximation; SRMR, standardized root mean square residual; CI, confidence interval.

### 3.4. Internal consistency and test-retest reliability

The Cronbach’s α coefficients of the 27-item OAS and its three factors are all above 0.8 (total: 0.913; Factor 1: 0.865; Factor 2: 0.838; Factor 3: 0.802). We used Pearson correlation coefficients and ICC to measure test-retest reliability (with a one-week interval). The results showed that the Pearson correlation coefficients of the OAS and its three factors were all significant and greater than or equal to 0.8 (total: 0.940; Factor 1: 0.831; Factor 2: 0.875; Factor 3: 0.800). The ICCs were all significant and greater than 0.75 (total: 0.940; Factor 1: 0.830; Factor 2: 0.874; Factor 3: 0.799) (as shown in Table 3).

**Table 3:** Reliability of the Chinese version of the OAS.

	Internal consistency	Test-retest reliability	
	Cronbach’s α	*r*	ICC
OAS	0.913	0.940^**^	0.940^**^
Factor 1	0.865	0.831^**^	0.830^**^
Factor 2	0.838	0.875^**^	0.874^**^
Factor 3	0.802	0.800^**^	0.799^**^

*r*, Pearson correlation coefficient; ICC, intraclass correlation coefficient. ^**^p < 0.01.

### 3.5. Concurrent validity

We utilized the Pearson correlation coefficient to explore the relationship between the OAS scores and those of other questionnaires. As indicated in the table, the total OAS score and its three sub-factors showed a significant positive correlation with the scores on BOSQ (*r* = 0.326–0.416, *p* < 0.01). The OAS score also positively correlated with the scores on GAD-7 (*r* = 0.116-0.155, *p* < 0.01). However, a negative correlation between the total OAS score and the scores on TAS-20 (*r* = −0.081, *p* < 0.05) was observed, as well as a significant negative correlation between Factor 1 and TAS-20 (*r* = −0.098, *p* < 0.01). A significant negative correlation was also found between Factor 3 and TAS-20 (*r* = −0.070, *p* < 0.05). Although a negative correlation between Factor 2 and TAS-20 was observed, it was not statistically significant (*r* = −0.046, *p* > 0.05) (see Table 4).

**Table 4:** Correlations between OAS and other related assessments.

	BOSQ	TAS-20	GAD-7	Factor 1	Factor 2	Factor 3	OAS
BOSQ	1						
TAS-20	0.061	1					
GAD-7	0.126^**^	0.323^**^	1				
Factor 1	0.373^**^	−0.098^**^	0.116^**^	1			
Factor 2	0.356^**^	−0.046	0.147^**^	0.568^**^	1		
Factor 3	0.326^**^	−0.070^*^	0.127^**^	0.630^**^	0.548^**^	1	
OAS	0.416^**^	−0.081^*^	0.155^**^	0.852^**^	0.880^**^	0.801^**^	1

OAS, Odor Awareness Scale; BOSQ, Body Odor Sniffing Questionnaire; TAS-20, Toronto Alexithymia Scale; GAD-7, Generalized Anxiety Disorder Scale. ^*^p < 0.05, ^**^p < 0.01.

## 4. Discussion

In the present study, we investigated the psychometric properties of the OAS in the context of Chinese medical students, with the main objective of exploring the factor structure of the scale. Through factor analysis, we identified three distinct factors, deviating from the original version’s two factors (9) and the single dimension found in the Spanish version (18). Collectively, our findings confirm the Chinese adaptation of the OAS as possessing adequate reliability and validity.

To ensure the questionnaire’s psychometric properties, it is necessary to perform item analysis as an initial test on the entire sample before conducting in-depth analysis. As a result, two items (i.e., item 31 and item 32) were removed because of their low total-item correlation coefficients, indicating that the consistency of these items with other items is not high, and the corresponding contents cannot be well reflected, which may be due to cultural differences, and may also be caused by the original scale that did not implement item analysis (9). During the EFA on sample 1, we compared two-, three-, four-, and five-factor structures. Because scale revision should retain original scale items to the maximum extent in accordance with the statistical principle, we finally chose the 3-factor model, which only needs to exclude one item to reach the set entry screening criteria. Factor naming is subjective to a certain extent and ultimately depends on the researcher’s definition (51). Our three-factor structure is different from the two-factor structure of the original scale, so we used different criteria to name the factors. After reviewing all items measuring the corresponding factors, Factor 1 was named “odor sensitivity,” referring to the ability to detect and differentiate various odors. Factor 2 was named “odor impact,” capturing the effect of odors on emotions, behaviors, and cognition. Factor 3 was named “odor attention,” involving attention to environmental odors. The original OAS did not conduct EFA (9), and the Spanish version only extracted one factor, explaining 25.87% of the cumulative variance in EFA (18), which is relatively low. In comparison, our revised Chinese version extracted three factors during the EFA stage, explaining 43.774% of the cumulative variance, indicating better psychometric properties and more information extracted from the questionnaire than the Spanish version.

We conducted a second-order CFA on this three-factor structure in sample 2, which is a more comprehensive and complete model than a first-order one (52). In the model modification indices section, not only the Chi-square values but also in contents we found that item 24 (“People differ in their sensitivity for odors. An unpleasant smell can leave one person unaffected yet unbearable to another. How sensitive to odors do you think you are?”) reflects the content of Factor 1 (odor sensitivity) more, while item 17 (“Do odors revive strong or vivid memories in you?”) reflects more of Factor 2 (odor impact). Therefore, Combining theory and data performance, we adjusted their positions and ultimately obtained a satisfactory fit index. The second-order CFA has been validated, indicating that three first-order factors load onto a higher-order factor (i.e., OAS), clarifying the relationships between the three factors and making the overall model more parsimonious, which is more consistent with the theoretical hypothesis (52, 53). At last, the revised OAS scale contains three factors with 27 items, including odor sensitivity with 8 items (items 7, 8, 9, 10, 11, 12, 13, 24), odor impact with 13 items (items 16, 17, 18, 19, 20, 21, 22, 23, 26, 27, 28, 29, 30), and odor attention with 6 items (items 1, 2, 3, 4, 5, 6). The original OAS separately subjected two factors to CFA rather than analyzing the two factors together (9), and thus, whether the OAS is suitable for a one-dimensional or two-dimensional solution or other multi-dimensional solutions is unclear. And the Spanish version did not perform CFA (18). In contrast, Our revised Chinese version of OAS involved analyzing the entire three-factor scale obtained through EFA in CFA, which yielded satisfactory results and addressed other versions’ shortcomings.

The overall Chinese version of the OAS has a Cronbach’s α coefficient of 0.913, and Cronbach’s α coefficients for the three factors are all above 0.8, indicating good internal consistency of the scale. The original version of the OAS did not measure Cronbach’s α coefficient for all items of the OAS but only measured Cronbach’s α coefficient for two factors (9), indicating that our revised scale has more integrity and internal consistency by comparison. The one-dimensional Spanish version of the OAS has a Cronbach’s α coefficient of 0.906 (18), which is comparable to our revised Chinese version. Concerning test-retest reliability, the results showed that the Pearson correlation coefficient was 0.940, and these for the three factors were all not less than 0.8; the ICC of the OAS was 0.94, and these for the three factors were all greater than 0.75, suggesting that the measurement stability and reliability of the OAS are sufficient. The original version of the OAS did not measure test-retest reliability (9), while the Spanish version used ICC to measure test-retest reliability, with a result of 0.895 (18), marginally lower than our revised Chinese version. The data suggests a slight superiority in the stability of the Chinese adaptation.

The results of the concurrent validity test showed the Chinese version of the OAS total score and the scores of the three factors were significantly positively correlated with the scores of BOSQ, indicating that the Chinese version of the OAS is also a psychometric scale closely related to odor. Furthermore, we found a significant positive correlation between the OAS total score and the scores of the three factors and GAD-7, consistent with previous research (17). In addition, some researchers have revealed that patients with panic disorder have a higher level of odor awareness than healthy individuals (28, 29), indicating a close relationship between odor awareness and anxiety symptoms. Our study results also showed a significant negative correlation between the OAS total score and the alexithymia scale score, consistent with previous research (17). Factors 1 and 3 also showed significant negative correlations with alexithymia, but Factor 2 did not have a significant correlation with alexithymia, although it showed a negative correlation. Dal Bo et al. (17) found that the interaction between social anxiety symptoms and alexithymia significantly predicted OAS scores, indicating that social anxiety symptoms moderated the relationship between OAS and alexithymia. Factor 2 (odor impact) refers to the effect of odors on individual emotions, behaviors, and cognition. The lack of a significant relationship between Factor 2 and alexithymia may be influenced by certain moderating factors, such as social anxiety, which requires further investigation in future research.

The relationship between olfaction and affect has been confirmed by many studies (20–22), and there is a physiological basis for their association, namely, the processing of olfactory and emotional signals by the brain’s limbic system (23). Based on the neurophysiological structure, many studies have corroborated the relationship between olfaction dysfunction and affective disorders, including depression, anxiety, and others (24–27). A study has revealed that individuals with anxiety disorders performed poorer olfactory discrimination compared to their healthy counterparts (54). Besides, research on the relationship between odor awareness and anxiety disorders has also obtained preliminary results (17, 28, 29). In contrast, depression symptoms positively predicted social odor awareness which refers to odor awareness related to body odor (15, 17). Social odor awareness reflects the individual’s social adaptation to some extent. And some studies have suggested that maladaptive social patterns are risk factors for the development of depression (55, 56). The association between odor awareness scale and depression should be implemented considering that many other previous studies reported similar correlations between olfactory dysfunction and depression levels (57, 58).

This study also has some limitations. The simple sampling method used to recruit university students limits the generalizability of the findings to a wider population, and the revised scale may have limited efficacy for other more diverse populations. Future studies could examine the reliability and validity of the OAS in different populations. Additionally, the little lower number of male participants than female participants may reduce the generalizability of the scale, and future research should aim to improve the balance of gender representation in a wider and more diverse population. However, the absolute number of male participants in this study was not small, and the scale can still apply to adult males. Compared to the original 32-item scale, the revised Chinese version of the OAS removes five items, resulting in a reduction of olfactory content. But the reliability and validity of the scale are improved and the remaining 27 items are more convenient to administer, which demonstrates it is a practical and valuable psychometric tool.

## 5. Conclusion

The results of the current study on Chinese medical students have revealed that the Chinese version of the OAS displays three first-order factors, which load onto a second-order factor, demonstrating good psychometric properties. The Chinese version of the OAS is a reliable and valid instrument for measuring odor awareness in Chinese people. It might be applicable to screen for affective symptoms, which is a new and valuable area for researchers to explore in depth. And also, the OAS is beneficial to the study of the relationship between olfactory performance and affective disorders.

## Data availability statement

The raw data supporting the conclusions of this article will be made available by the authors, without undue reservation.

## Ethics statement

The studies involving human participants were reviewed and approved by the Ethics Committee of Guangxi Medical University. The patients/participants provided their written informed consent to participate in this study.

## Author contributions

BZ, XL, and PT contributed to the conception, design of the study, and performed the statistical analysis. YL, WH, LW, and SH collected data and conducted research. BZ wrote the first draft of the manuscript. GN, XL, and PT revised the manuscript. GN had primary responsibility for the final content. All authors contributed to the article and approved the submitted version.

## Funding

This work was supported by Humanities and Social Sciences Project of the Training Program for Thousands of Young and Middle-aged Key Teachers in Guangxi [grant number 2020QGRW010].

## Conflict of interest

The authors declare that the research was conducted in the absence of any commercial or financial relationships that could be construed as a potential conflict of interest.

## Publisher’s note

All claims expressed in this article are solely those of the authors and do not necessarily represent those of their affiliated organizations, or those of the publisher, the editors and the reviewers. Any product that may be evaluated in this article, or claim that may be made by its manufacturer, is not guaranteed or endorsed by the publisher.

## References

[1] Baron RA. Environmentally induced positive affect: its impact on self-efficacy, task performance, negotiation, and conflict 1. J Appl Soc Psychol. (1990) 20:368–84. doi: 10.1111/j.1559-1816.1990.tb00417.x

[2] Ehrlichman H Bastone L. The use of odour in the study of emotion. Fragrance: The psychology and biology of perfume. New York, NY, US: Elsevier Applied Science Publishers/Elsevier Science Publishers (1992) 143–59.

[3] Wrzesniewski A McCauley C Rozin P. Odor and affect: individual differences in the impact of odor on liking for places, things and people. Chem Senses. (1999) 24:713–21. doi: 10.1093/chemse/24.6.713, PMID: 10587506

[4] Fiore AM Yah X Yoh E. Effects of a product display and environmental fragrancing on approach responses and pleasurable experiences. Psychol Mark. (2000) 17:27–54. doi: 10.1002/(SICI)1520-6793(200001)17:1<27::AID-MAR3>3.0.CO;2-C

[5] Larsson M Willander J. Autobiographical odor memory. Ann N Y Acad Sci. (2009) 1170:318–23. doi: 10.1111/j.1749-6632.2009.03934.x 19686154

[6] KRM RAO ER JETTI. Scent marketing–harnessing the power of scents in stimulating senses of organized retail consumers and employees. Group. (2020) 1362:60.53. doi: 10.38124/IJISRT20SEP134

[7] Berčík J Neomániová K Mušinská K Pšurný M. Use of consumer neuroscience in the choice of aromatisation as part of the shopping atmosphere and a way to increase sales volume. Appl Sci. (2022) 12:7069. doi: 10.3390/app12147069

[8] White TL Cunningham C. Sexual preference and the self-reported role of olfaction in mate selection. Chemosens Percept. (2017) 10:31–41. doi: 10.1007/s12078-017-9223-9

[9] Smeets MA Schifferstein HN Boelema SR Lensvelt-Mulders G. The odor awareness scale: a new scale for measuring positive and negative odor awareness. Chem Senses. (2008) 33:725–34. doi: 10.1093/chemse/bjn038, PMID: 18622009

[10] Nováková L Varella Valentova J Havlíček J. Engagement in olfaction-related activities is associated with the ability of odor identification and odor awareness. Chemosens Percept. (2014) 7:56–67. doi: 10.1007/s12078-014-9167-2

[11] Oleszkiewicz A Alizadeh R Altundag A Chen B Corrai A Fanari R. Global study of variability in olfactory sensitivity. Behav Neurosci. (2020) 134:394–406. doi: 10.1037/bne0000378, PMID: 33001681

[12] Cupchik G Phillips K Truong H. Sensitivity to the cognitive and affective qualities of odours. Cognit Emot. (2005) 19:121–31. doi: 10.1080/0269993044100011

[13] Ferdenzi C Coureaud G Camos V Schaal B. Human awareness and uses of odor cues in everyday life: results from a questionnaire study in children. Int J Behav Dev. (2008) 32:422–31. doi: 10.1177/0165025408093661

[14] Martin GN Apena F Chaudry Z Mulligan Z Nixon C. The development of an attitudes towards the sense of smell questionnaire (SoSQ) and a comparison of different Professions' responses. North. Am J Psychol. (2001) 3:491–502. Available at: https://eprints.mdx.ac.uk/3516/

[15] Dal Bo E Gentili C Spoto A Bruno G Castellani A Tripodi C. The social odor scale: development and initial validation of a new scale for the assessment of social odor awareness. PLoS One. (2021) 16:e0260587. doi: 10.1371/journal.pone.0260587, PMID: 34905551 PMC8670672

[16] Li ZL Yue Q Mahmut MK Zou LQ. Do you often sniff yourself or others? Development of the body odor sniffing questionnaire and a cross-cultural survey in China and the USA. Physiol Behav. (2022) 255:113934. doi: 10.1016/j.physbeh.2022.113934 35908610

[17] Dal Bo E Gentili C Castellani A Tripodi C Fischmeister FPS Cecchetto C. Olfactory meta-cognition in individuals with depressive and anxiety symptoms: the differential role of common and social odors. J Affect Disord. (2022) 308:259–67. doi: 10.1016/j.jad.2022.04.071, PMID: 35429542

[18] Buron E Bulbena A Pailhez G Bulbena CA. The Spanish version of two olfactory scales: reliability and validity. Rev Psiquiatr Salud Ment. (2011) 4:187–94. doi: 10.1016/j.rpsmen.2011.12.001 23446264

[19] Arshamian A Willander J Larsson M. Olfactory awareness is positively associated to odour memory. J Cogn Psychol. (2011) 23:220–6. doi: 10.1080/20445911.2011.483226

[20] Lane RD Reiman EM Bradley MM Lang PJ Ahern GL Davidson RJ. Neuroanatomical correlates of pleasant and unpleasant emotion. Neuropsychologia. (1997) 35:1437–44. doi: 10.1016/S0028-3932(97)00070-5, PMID: 9352521

[21] Atanasova B El-Hage W Chabanet C Gaillard P Belzung C Camus V. Olfactory anhedonia and negative olfactory alliesthesia in depressed patients. Psychiatry Res. (2010) 176:190–6. doi: 10.1016/j.psychres.2008.11.016, PMID: 20207422

[22] Zald DH Pardo JV. Functional neuroimaging of the olfactory system in humans. Int J Psychophysiol. (2000) 36:165–81. doi: 10.1016/S0167-8760(99)00110-5 10742571

[23] Soudry Y Lemogne C Malinvaud D Consoli SM Bonfils P. Olfactory system and emotion: common substrates. Eur Ann Otorhinolaryngol Head Neck Dis. (2011) 128:18–23. doi: 10.1016/j.anorl.2010.09.007 21227767

[24] Kamath V Paksarian D Cui L Moberg PJ Turetsky BI Merikangas KR. Olfactory processing in bipolar disorder, major depression, and anxiety. Bipolar Disord. (2018) 20:547–55. doi: 10.1111/bdi.12625 29441710

[25] Kohli P Soler ZM Nguyen SA Muus JS Schlosser RJ. The association between olfaction and depression: a systematic review. Chem Senses. (2016) 41:479–86. doi: 10.1093/chemse/bjw061, PMID: 27170667 PMC4918728

[26] Mattos JL Schlosser RJ Storck KA Soler ZM. Understanding the relationship between olfactory-specific quality of life, objective olfactory loss, and patient factors in chronic rhinosinusitis. Int Forum Allergy Rhinol. (2017) 7:734–40. doi: 10.1002/alr.21940, PMID: 28519966 PMC5751751

[27] Taalman H Wallace C Milev R. Olfactory functioning and depression: a systematic review. Front Psych. (2017) 8:190. doi: 10.3389/fpsyt.2017.00190, PMID: 29033860 PMC5627007

[28] Buron E Bulbena A Bulbena-Cabre A. Olfactory functioning in panic disorder. J Affect Disord. (2015) 175:292–8. doi: 10.1016/j.jad.2015.01.049, PMID: 25661394

[29] Buron E Bulbena A Bulbena-Cabre A Rosado S Pailhez G. Both anxiety and joint laxity determine the olfactory features in panic disorder. Psychiatry Res. (2018) 262:420–6. doi: 10.1016/j.psychres.2017.09.018, PMID: 28923431

[30] Koelega HS. Sex differences in olfactory sensitivity and the problem of the generality of smell acuity. Percept Mot Skills. (1994) 78:203–13. doi: 10.2466/pms.1994.78.1.203, PMID: 8177660

[31] Pause BM Adolph D Prehn-Kristensen A Ferstl R. Startle response potentiation to chemosensory anxiety signals in socially anxious individuals. Int J Psychophysiol. (2009) 74:88–92. doi: 10.1016/j.ijpsycho.2009.07.008, PMID: 19666058

[32] Chen D Dalton P. The effect of emotion and personality on olfactory perception. Chem Senses. (2005) 30:345–51. doi: 10.1093/chemse/bji029 15788711

[33] Havlícek J Nováková L Vondrová M Kubena AA Valentová J Roberts SC. Olfactory perception is positively linked to anxiety in young adults. Perception. (2012) 41:1246–61. doi: 10.1068/p7244, PMID: 23469704

[34] Krusemark EA Li W. Enhanced olfactory sensory perception of threat in anxiety: an event-related fMRI study. Chemosens Percept. (2012) 5:37–45. doi: 10.1007/s12078-011-9111-7, PMID: 22866182 PMC3410736

[35] Buron E Bulbena A Barrada JR Pailhez G. EROL scale: a new behavioural olfactory measure and its relationship with anxiety and depression symptoms. Actas Esp Psiquiatr. (2013) 41:2–9. PMID: 23440530

[36] Bagby RM Parker JD Taylor GJ. The twenty-item Toronto alexithymia scale--I. item selection and cross-validation of the factor structure. J Psychosom Res. (1994) 38:23–32. doi: 10.1016/0022-3999(94)90005-1, PMID: 8126686

[37] Jinyao Y Shuqiao Y Xiongzhao Z. The Chinese version of the TAS-20: reliability and validity. Chin Ment Health J. (2003) 17:763–7. Available at: https://psycnet.apa.org/record/2003-10252-010

[38] Spitzer RL Kroenke K Williams JB Löwe B. A brief measure for assessing generalized anxiety disorder: the GAD-7. Arch Intern Med. (2006) 166:1092–7. doi: 10.1001/archinte.166.10.1092 16717171

[39] Xiaoyan H Chunbo L Jie Q Haisong C Wenyuan W. Reliability and validity of a generalized anxiety disorder scale in general hospital outpatients. Shanghai Arch Psychiatry. (2010) 22:200–3. doi: 10.3969/j.issn.1002-0829.2010.04.002

[40] Nunnally JC Bernstein IH. Psychometric Theory. 3rd ed. New York: McGraw-Hill (1994).

[41] Barrientos-Trigo S Gil-García E Romero-Sánchez J Badanta-Romero B Porcel-Gálvez A. Evaluation of psychometric properties of instruments measuring nursing-sensitive outcomes: a systematic review. Int Nurs Rev. (2019) 66:209–23. doi: 10.1111/inr.12495, PMID: 30378685

[42] Yong AG Pearce S. A beginner’s guide to factor analysis: focusing on exploratory factor analysis. Tutorials in quantitative methods for psychology. (2013) 9:79–94. doi: 10.20982/tqmp.09.2.p079

[43] Hair JF. Multivariate data analysis. Kennesaw: Faculty and Research Publications (2009).

[44] Corner S. Choosing the right type of rotation in PCA and EFA. JALT testing & evaluation SIG newsletter. (2009) 13:20–5. Available at: https://hosted.jalt.org/test/bro_31.htm

[45] Costello AB Osborne J. Best practices in exploratory factor analysis: four recommendations for getting the most from your analysis. Pract Assess Res Eval. (2005) 10:7. doi: 10.7275/jyj1-4868

[46] Kline P. An Easy Guide to Factor Analysis. London: Routledge (2014).

[47] Byrne BM. Structural Equation Modeling With EQS: Basic Concepts, Applications, and Programming, Second Edition. London: Routledge (2013).

[48] Schermelleh-Engel K Moosbrugger H Müller H. Evaluating the fit of structural equation models: tests of significance and descriptive goodness-of-fit measures. Methods Psychol Res Online. (2003) 8:23–74. Available at: https://psycnet.apa.org/record/2003-08119-003

[49] Lt H Bentler PM. Cutoff criteria for fit indexes in covariance structure analysis: conventional criteria versus new alternatives. Struct Equ Model Multidiscip J. (1999) 6:1–55. doi: 10.1080/10705519909540118

[50] Tabachnick BG Fidell LS Ullman JB. Using multivariate statistics. Boston: Pearson(2013).

[51] Henson RK Roberts JK. Use of exploratory factor analysis in published research. Educ Psychol Meas. (2016) 66:393–416. doi: 10.1177/0013164405282485

[52] Chen FF Sousa KH West SG. Teacher's Corner: testing measurement invariance of second-order factor models. Struct Equ Model Multidiscip J. (2005) 12:471–92. doi: 10.1207/s15328007sem1203_7

[53] Brown TA. Confirmatory factor analysis for applied research. New York: Guilford publications (2015).

[54] Clepce M Reich K Gossler A Kornhuber J Thuerauf N. Olfactory abnormalities in anxiety disorders. Neurosci Lett. (2012) 511:43–6. doi: 10.1016/j.neulet.2012.01.034 22306090

[55] Slavich GM O'Donovan A Epel ES Kemeny ME. Black sheep get the blues: a psychobiological model of social rejection and depression. Neurosci Biobehav Rev. (2010) 35:39–45. doi: 10.1016/j.neubiorev.2010.01.003, PMID: 20083138 PMC2926175

[56] Coyne JC. Toward an interactional description of depression. Psychiatry. (1976) 39:28–40. doi: 10.1080/00332747.1976.11023874 1257353

[57] Croy I Nordin S Hummel T. Olfactory disorders and quality of life--an updated review. Chem Senses. (2014) 39:185–94. doi: 10.1093/chemse/bjt072, PMID: 24429163

[58] Sanna F Loy F Piras R Moat A Masala C. Age-related cognitive decline and the olfactory identification deficit are associated to increased level of depression. Front Neurosci. (2021) 15:599593. doi: 10.3389/fnins.2021.599593 33692667 PMC7937898

