# Supplementary material for: Factor structure and psychometric properties of the Chinese version of the Odor Awareness Scale
Source: Front Psychiatry. 2023 Jul 27;14:1228179. doi: 10.3389/fpsyt.2023.1228179 (PMC10415028; doi:10.3389/fpsyt.2023.1228179)
Supplement: Supplementary file 1 [file Table_1.DOCX]

Supported information

Items of the OAS in English

| 1. | When you walk through the woods, do you pay attention to the odors surrounding you? |
| --- | --- |
| 2. | When someone is busy in the kitchen, do you notice the odor of the food being prepared? |
| 3. | Do you notice food odors emanating from houses when you are outdoors? |
| 4. | When you are studying, or concentrated in general, do you get distracted by odors in the environment? |
| 5. | When you visit someone else’s house, do you notice how it smells? |
| 6. | Do you sniff at a new book? |
| 7. | When an acquaintance smells differently from normal, for example, because of a new perfume, do you immediately notice? |
| 8. | Do you notice the smell of people’s breath or sweat? |
| 9. | Do you pay attention to the perfume, the aftershave or deodorant other people use? |
| 10. | Are you the first one to smell gas? |
| 11. | Are you the first one to smell when the milk is sour? |
| 12. | Are you the first one to smell a fire, even when the smell only comes from a barbecue or fireplace? |
| 13. | Are you the first one to smell spoilt food in the fridge? |
| 16. | Does an unpleasant smell in the environment that won’t go away make you anxious? |
| 17. | Do odors revive strong or vivid memories in you? |
| 18. | Do you sniff at clothes before you put them on? |
| 19. | The smell of smoke or food is still lingering in your clothes from the night before. Do you put on new clothes because of the smell? |
| 20. | Does the smell of food sometimes put you off it? |
|  | Response category used for items 1-13 and items16-20: Always (5), often (4), sometimes (3), seldom (2), never (1) |
| 21. | When a room has an unpleasant smell, does it influence your mood? |
| 22. | When someone has an unpleasant body odor, does that make you find him or her unattractive? The body odor |
| 23. | When someone has a pleasant body odor, do you find him or her attractive? The body odor_____ |
|  | Response category used for items 21-23: Has very much influence (5), much influence (4), some influence (3), a little influence (2), (almost) no influence (1) |
| 24. | People differ in their sensitivity for odors. An unpleasant smell can leave one person unaffected yet be unbearable to another. How sensitive to odors do you think you are?" |
|  | Response category for item 24: Much more sensitive than others (5), more sensitive than others (4), equally sensitive as others (3), less sensitive than others(2), much less sensitive than others (1) |
| 26. | How important is it to you that your sheets smell fresh? |
| 27. | How important is it to you that your (future/potential) partner has a pleasant smell? |
| 28. | Nowadays many cultivated flowers no longer have a fragrance. Do you find it important that flowers are fragrant? |
| 29. | How important are odors to you in your everyday life? |
|  | Response category for 26–29: Very important to not important at all, on a 5-point scale |
| 30. | You are in a public space sitting close to someone who has an unpleasant smell. Do you look for another seat if possible? |
|  | Response category to be used: Yes (5), probably (4), perhaps (3), probably not (2), no (1) |
| Three subscales: | |
| Odor Sensitivity: referring to the ability to detect and differentiate various odors with 8 items (items 7, 8, 9, 10, 11, 12, 13, 24). | |
| Odor Impact: referring to the effect of odors on emotions, behaviors and cognition with 13 items (items 16, 17, 18, 19, 20, 21, 22, 23, 26, 27, 28, 29, 30). | |
| Odor Attention: referring to the attention to environmental odors with 6 items (items 1, 2, 3, 4, 5, 6). | |

Items of the OAS in Chinese

| 1. | 当您穿过树林时，您会注意到周围的气味吗? |
| --- | --- |
| 2. | 当有人在厨房里忙碌时，您会注意到正在准备的食物的气味吗? |
| 3. | 当您在屋子外面时，您会注意到从屋子里散发出来的食物气味吗? |
| 4. | 通常您在学习或集中注意力时，您会被环境中的气味分散注意力吗? |
| 5. | 当您去别人家里时，您会注意到房子里的气味吗? |
| 6. | 您会闻一本新书的气味吗？ |
| 7. | 当一个熟人的气味与平常不同时，例如一种新香水味，您会立即注意到吗? |
| 8. | 您会注意到人们呼吸或汗水的气味吗? |
| 9. | 您会关注别人用的香水或体香剂吗? |
| 10. | 您是最先闻到环境中煤气味的人吗? |
| 11. | 您是最先闻到牛奶变酸臭的人吗? |
| 12. | 您是最先闻到物体燃烧气味的人吗，即使这种气味只来自烧烤架或壁炉? |
| 13. | 您是最先闻到冰箱里食物变质的人吗? |
| 16. | 环境中挥之不去的难闻气味会让您焦虑吗? |
| 17. | 气味会唤醒您强烈或生动的回忆吗? |
| 18. | 您穿衣之前会闻一下衣服吗? |
| 19. | 前一晚的烟味和食物气味还残留在您的衣服上，您会因此而换衣服吗? |
| 20. | 食物的气味有时会让您失去对它的兴趣吗? |
|  | 条目1-13，16-20的选项为：总是（5），经常（4），有时（3），很少（2），从不（1） |
| 21. | 当房间里有难闻的气味时，它会影响您的心情吗? |
| 22. | 当一个人有难闻的体味时，您会觉得他或她没有吸引力吗? 体味______________ |
| 23. | 当一个人有好闻的体味时，您觉得他/她有吸引力吗? 体味______________ |
|  | 条目21-23的选项为：非常有影响（5），很有影响（4），有一些影响（3），有一点影响（2），(几乎)没有影响（1） |
| 24. | 人们对气味的敏感度各不相同。对于难闻的气味，有些人不受影响，但有些人却无法忍受。您认为您对气味有多敏感? |
|  | 条目24的选项为：比别人敏感得多（5），比别人更敏感（4），跟别人一样敏感（3），不如别人敏感（2），远不如别人敏感（1） |
| 26. | 您的床单闻起来清新，这对您有多重要? |
| 27. | 您的(未来/潜在的)伴侣有好闻的气味对您来说有多重要? |
| 28. | 现在许多栽培的花已经没有香味了。您觉得花有香味很重要吗? |
| 29. | 气味在您的日常生活中有多重要? |
|  | 条目26-29的选项为：非常重要（5），比较重要（4），一般（3），比较不重要（2），完全不重要（1） |
| 30. | 在公共场合，您坐在一个有难闻气味的人旁边。如果可能的话，您会找另一个座位吗？ |
|  | 条目30的选项为：会（5），很可能会（4），可能会（3），很可能不会（2），不会（1） |
